# A Longitudinal Study of Predictors for Adolescent Electronic Cigarette Experimentation and Comparison with Conventional Smoking

**DOI:** 10.3390/ijerph15020305

**Published:** 2018-02-09

**Authors:** Jaana M. Kinnunen, Hanna Ollila, Jaana Minkkinen, Pirjo L. Lindfors, Arja H. Rimpelä

**Affiliations:** 1 Faculty of Social Sciences, Health Sciences, University of Tampere, 33014 Tampere, Finland; jaana.minkkinen@uta.fi (J.M.); pirjo.lindfors@uta.fi (P.L.L.); arja.rimpela@staff.uta.fi (A.H.R.); 2 Alcohol, Drugs and Addictions Unit, Department of Public Health Solutions, National Institute for Health and Welfare, 00271 Helsinki, Finland; hanna.ollila@thl.fi; 3 PERLA—Tampere Centre for Childhood, Youth and Family Research, University of Tampere, 33014 Tampere, Finland; 4 Department of Adolescent Psychiatry, Pitkäniemi Hospital, Tampere University Hospital, 33380 Nokia, Finland

**Keywords:** electronic cigarette, adolescents, smoking, predictors, school survey

## Abstract

Little is known of the predictors of electronic cigarette (e-cigarette) use among adolescents, even though the use is increasing. We studied here the predictors for e-cigarette experimentation (tried and tried more than twice) and compared them with predictors for conventional smoking. A baseline school survey was conducted in the Helsinki metropolitan area, Finland, in 2011 for seventh graders (12 to 13-year-olds). Response rate was 73%. The same students were followed up in 2014 (9th grade, 15 to 16-year-olds), *N* = 5742. Generalized linear mixed models controlling for school clustering were used. In the follow-up, 43.3% of boys and 25.6% of girls had tried e-cigarettes and 21.9% and 8.1% correspondingly more than twice. The strongest predictors for both genders were conventional smoking, drunkenness and energy drink use. Furthermore, poor academic achievement predicted e-cigarette experimentation for both genders, and for boys, participation in team sports was a predictor. The predictors for experimenting and for experimenting more than twice were very similar, except for boys’ participation in team sports. They were also similar compared to the predictors of conventional smoking but the associations were weaker. To conclude, smoking and other addictive behaviors predict adolescents’ experimentation with e-cigarettes. Family’s socioeconomic background had little significance.

## 1. Introduction

Electronic cigarettes (e-cigarettes) or electronic nicotine delivery systems (ENDS) have taken root all over the world among younger populations during the last few years [1,2,3,4]. Young smokers seem to be more prone to experiment with and use e-cigarettes, but also among those who have never tried smoking, e-cigarette experimentation has been reported [5,6,7]. As e-cigarette experimentation has increased rapidly among adolescents and e-cigarettes may also be a gateway to conventional smoking [8], curbing the increase in adolescents’ e-cigarette experimentation should be promoted. To identify the susceptible adolescents better, the risk factors for e-cigarette experimentation need to be studied in detail.

The correlates for adolescent e-cigarette experimentation and use have been studied quite widely in cross-sectional studies. So far, adolescent e-cigarette experimentation and use have been associated with other addictive behaviors: susceptibility to conventional smoking initiation [6,9], conventional smoking [4,5,10,11,12,13,14,15,16,17,18,19,20,21], ever-use of other tobacco products (combustible and non-combustible) [10,12,14,18], and alcohol [4,11,14,22] and cannabis use [20,21]. Additionally, male gender [4,11,13,14,16,22,23], perception of low harm of e-cigarettes [24,25,26,27], peer smoking [4,13,20,26], parents’ smoking [10,18,23] and exposure to e-cigarette advertising [28,29] have been associated with e-cigarette use.

There are only a handful of longitudinal studies on adolescent e-cigarette use that have been published so far, and they have concentrated on the progression to conventional cigarette smoking after e-cigarette use [30,31,32,33,34,35] and on the predictors of continued e-cigarette use after experimentation [36]. To our knowledge, predictors for adolescent e-cigarette experimentation have been studied only once in a longitudinal setting. In this German study [37], sensation-seeking behavior and friends’ and parental smoking predicted e-cigarette use, while conventional cigarette smoking and male gender did not [37]. The above-mentioned study [37] also compared the risk factors between e-cigarette use and conventional cigarette smoking: some of the risk factors were the same but, e.g., male gender and older age predicted only conventional cigarette smoking. In Finnish studies, male gender has been associated with e-cigarette experimentation [10,18]. Thus, there is a need for gender-stratified and more detailed analyses of factors that increase the risk for e-cigarette experimentation and use.

This study explores predictors for e-cigarette experimentation separately for boys and girls in a longitudinal setting in Finland. As the risk factors for e-cigarette use and use of conventional cigarettes may be different [7], we compare the predictors for both. The predictors to be studied include addictive health behaviors (drunkenness, use of energy drinks), socioeconomic and family background, parents’ smoking, and student’s own academic achievement at school. These are known to be risk factors or correlates for smoking as well [38]. As smokeless tobacco (snus) use has been found to be associated with participation in team sports [39], three different kinds of leisure activities, namely participation in team sports, individual sports, and music, art or club activities were also included in the investigated predictors.

At the time of the surveys, there was no age limit in Finland for purchasing non-nicotine e-cigarettes as they were classified as tobacco imitations and e-liquids as substitute tobacco. The age limit for conventional cigarettes was 18 years. Nicotine-containing e-cigarettes at that time were treated as medicinal products and no e-cigarette company had a selling permit for them. However, e-cigarettes with nicotine were acquired from visits abroad or online also by adolescents, along with friends as a main source [10]. According to the new Tobacco Act of 2016, e-cigarettes, both nicotine and non-nicotine, are considered equal to conventional cigarettes [40]. 

## 2. Materials and Methods 

### 2.1. Participants and Study Procedure

**Figure 1:**
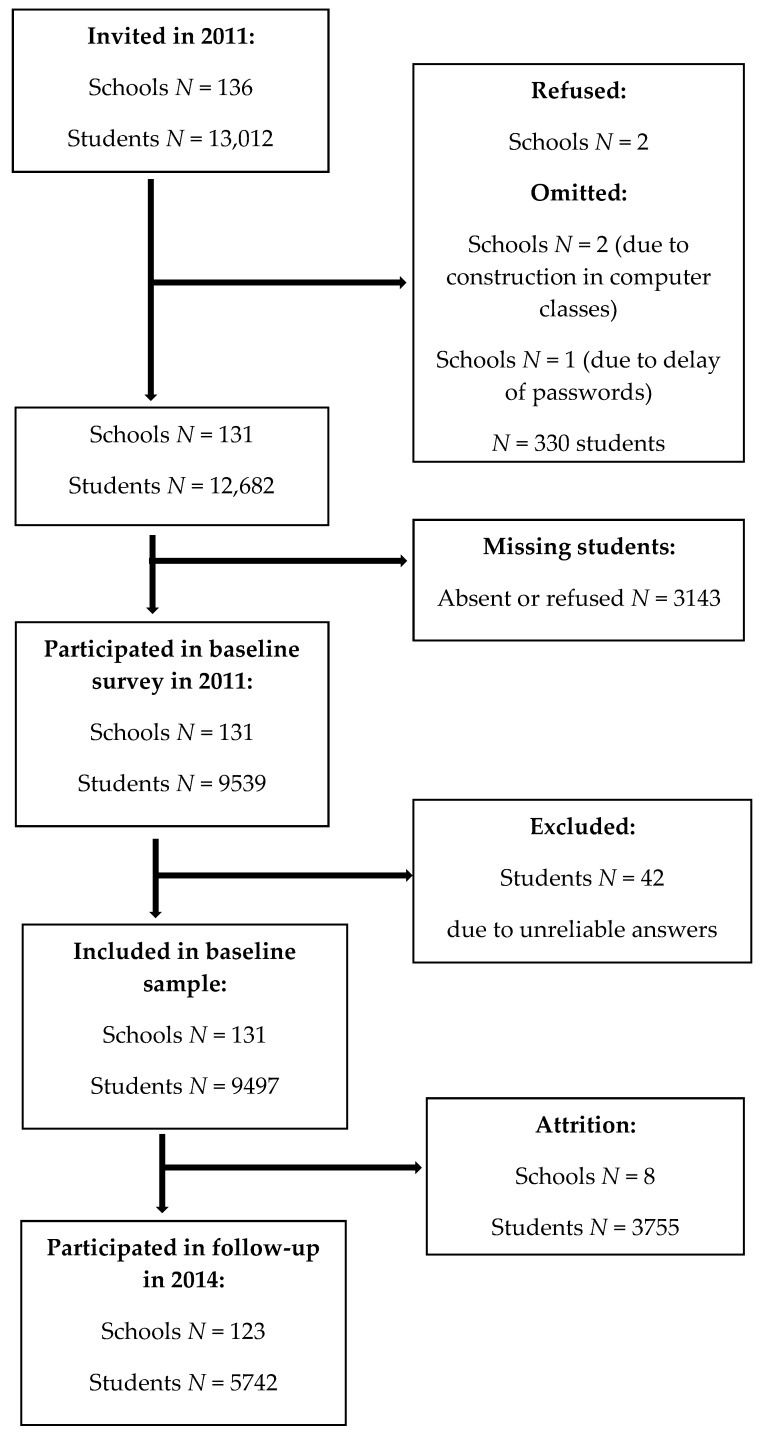
Flow diagram representing the formation of the study population.

Metropolitan Longitudinal Finland (MetLoFIN) is a longitudinal study following a cohort of 13,012 children in the Helsinki metropolitan area of Finland. The study covered all schools of 14 metropolitan municipalities (*N* = 136). The first school survey was conducted in autumn 2011 (baseline) in the beginning of the lower secondary school, i.e., 7th grade (12 to 13-year-olds), and 9497 people of the cohort participated in the survey, meaning a response rate of 73%. In the city of Helsinki, five schools (2.5%; *N* = 330) were omitted: two schools refused to participate, two schools had construction in their computer classes and one school had a delay of the individual passwords for the survey. Almost empty and unreliable questionnaires, i.e., profanities in open-ended questions and extreme choices, were excluded (*N* = 42). Additionally, some students were absent from the school on the survey day or refused to participate (separate N’s not known). The second survey was conducted in spring 2014 (follow-up) at the end of lower secondary school, i.e., 9th grade (15 to 16-year-olds). The flow diagram representing the formation of the study population is presented in Figure 1.

The data was gathered as part of the school routine and, therefore, no parental consent was needed according to the ethical guidelines of the Finnish Advisory Board on Research Integrity (www.tenk.fi/en). However, the local authorities required parental consents in two of the 14 municipalities and the consents were collected. In other municipalities, parents received information letters on the survey, and were able to deny their children the participation (=passive consent). The participants completed an online survey in computer classrooms using personal user names and passwords. The study protocol has been approved by the Ethics Committee of the Finnish National Institute of Health and Welfare. For the 2011 survey, the statement code is 27.5.2011 and the code for the 2014 survey is 9.4.2014.

The well-being survey consisted of questions regarding well-being, health, health behavior, school, and family background. E-cigarette use was assessed at follow-up, and predictors are analyzed from the baseline. E-cigarette use was not asked at baseline but it can be well assumed that the students were never-users of e-cigarettes or that there were just very few of them in 2011. The reason for this assumption is that, according to our other research, e-cigarette use was very rare among 12-year-olds in Finland after two years of the survey in 2013, and most of them had not even heard about e-cigarettes [10]. All respondents who answered both baseline and follow-up (*N* = 5742, *N* for schools = 123) were included in the analyses, meaning 60.5% of those who participated in the first survey and 44.1% of the original cohort. The distribution of boys and girls was equal (Boys: *N* = 2871; Girls: *N* = 2871). The descriptive statistics of the study population are presented in Table 1.

### 2.2. Measures

In 2014 (follow-up), e-cigarette use was asked with a question “Have you sometimes used the following products?” E-cigarettes with nicotine liquid and e-cigarettes with other liquid were asked separately. The options were ‘No’, ‘I have tried once or twice’, ‘I have used 20 times or less’ and ‘I have used more than 20 times’. For the analyses of experimentation, the questions were combined into a variable ‘Has tried an e-cigarette’ and it was dichotomized as ‘Not tried’ and ‘Tried’. For the analyses of use for more than twice, the questions were combined into a variable ‘Has tried an e-cigarette more than twice’ and it was dichotomized as ‘Not tried’ and ‘Tried’. The reason for combining the groups of different types of liquids was that most of the students who had tried e-cigarettes had tried both types of liquids (overlap in girls 441 and in boys 830 students). Additionally, whether the e-cigarette contains nicotine or not does not seem to be meaningful for the adolescents when they experiment with the product, and many of the adolescents do not even know about the contents of the e-liquid [18].

At baseline and follow-up, smoking experimentation was asked with a question: ”Have you ever smoked? If you have, how many cigarettes have you smoked altogether until now?” The options were ‘I have never tried smoking’, ‘One’, ‘About 2 to 50’ and ‘More than 50’. The variable ‘Tried smoking’ was dichotomized as ‘Not tried’ and ‘Tried’, and the variable ‘Smoked over 50 cigarettes’ was dichotomized as ‘Not smoked’ and ‘Smoked’. The inconsistent answers (*N* = 89) in smoking, i.e., reporting tried smoking at baseline but not tried at follow-up, were corrected so that the follow-up answer coincided with the baseline answer. List of the questions, answering options and created categories on baseline predictors can be found from Table A1. The proportion of missing answers was small for all variables (0.1–4.2%), except for parents’ education (proportion of missing answers 14%).

### 2.3. Attrition Analysis

To assess attrition, the students who answered both surveys (=sample in the analyses, *N* = 5742) were compared to those students who completed only the baseline survey but not the follow-up (=attrition, *N* = 3755) using some answers of the baseline survey. In the attrition, there were statistically significantly (*p* < 0.001) more students with poorer academic achievement (e.g., poor 9.3% and excellent 20.2%) compared to the sample (poor 5.9% and excellent 24.6%). The students in the attrition also had tried more smoking (*p* = 0.002; 23.4%) compared to the students in the sample (18.5%). There was no statistically significant difference in the gender distribution (*p* = 0.795), nor in the distribution of parental education (*p* = 0.099).

The distributions of these variables were also compared between the students in the final sample used in the analyses (*N* = 5742) and all the students who completed the questionnaire at baseline (*N* = 9497). The distributions of gender and parents’ education were very close to each other. However, students in the final sample in the analyses had better academic achievement (e.g., poor 5.9% and excellent 24.6%) compared to the original baseline sample (poor 7.2% and excellent 22.9%). Additionally, the final sample included more of those who had not tried smoking (81.5%) compared to the students who completed the questionnaire at baseline (79.5%).

### 2.4. Data Analysis

First, any e-cigarette experimentation and experimentation more than twice, and conventional cigarette experimentation and smoking at follow-up were cross-tabulated with all independent baseline variables separately for boys and girls (not shown in tables). Second, gender stratified multilevel binary logistic regression analyses were conducted to analyze predictors for any e-cigarette experimentation and experimentation more than twice, and for experimentation with cigarettes and smoking more than 50 cigarettes at follow-up for all independent baseline variables. Then, all statistically significant independent baseline variables were included in a multivariate logistic regression model. The multilevel logistic regression analyses were conducted with generalized linear mixed models (GLMM) with school as the random effect. The variances at school-level in follow-up e-cigarette and smoking questions were of small magnitude (1.2% to 1.7%) but statistically significant. The Test of independence in Complex Samples command, which takes the clustering into account, was used to test statistical differences. IBM SPSS Statistics V.23 (IBM, Armonk, NY, USA) was used for all data analyses.

## 3. Results

At follow-up in 2014, of all 15 to 16-year-old students, 34.3% had tried e-cigarettes, 43.3% of boys and 25.6% of girls (Table 1, *p* < 0.001 between genders). Conventional cigarette smoking had been tried at baseline by 21.8% of 12 to 13-year-old boys and 14.9% of girls in 2011 (*p* < 0.001), and by follow-up in 2014, 50.0% of boys and 42.3% of girls (*p* < 0.001) had tried conventional cigarette smoking (Table 1).

### 3.1. Predictors among Boys

In bivariate logistic regressions (Table 2), the strongest predictors for boys’ e-cigarette experimentation at follow-up were baseline addictive behavior factors: daily (OR 61.12; 95% CI 8.30–450.0), occasional (OR 15.58; 95% CI 7.05–34.45) and weekly conventional smoking (OR 9.60; 95% CI 3.64–25.30), drunkenness at least once (OR 7.08; 95% CI 4.92–10.21) and energy drink daily use (OR 6.70; 95% CI 4.63–9.69). Family background factors and academic achievement also predicted e-cigarette experimentation, with poor academic achievement as the strongest predictor (OR 3.63; 95% CI 2.54–5.19). Participation in leisure activities was mainly negatively associated with e-cigarette experimentation, with the exception of involvement in team sports (OR 1.43; 95% CI 1.23–1.67). The predictors for boys’ conventional smoking experimentation were rather similar but stronger, e.g., drunkenness at least once (OR 15.49; 95% CI 9.09–26.40), compared to the predictors for e-cigarette experimentation, except for leisure activities, of which only participation in music, art or club activities was negatively associated with smoking experimentation (Table 2).

In multivariate logistic regressions (Table 2), the most significant predictors for boys’ e-cigarette experimentation at follow-up were baseline daily (OR 19.26; 95% CI 2.51–147.7), occasional (OR 7.02; 95% CI 2.85–17.31) and weekly conventional smoking (OR 5.04; 95% CI 1.64–15.50). Participating in team sports was also a predictor for boys’ e-cigarette experimentation (OR 1.90; 95% CI 1.55–2.32). The strongest predictors for boys’ smoking experimentation in the multivariate model were drunkenness at least once (OR 9.41; 95% CI 5.23–16.95) and daily energy drink use (OR 6.27; 95% CI 3.65–10.77).

In analyses for e-cigarette experimentation more than twice (Table 3), the strongest risk factors from baseline were the same as for experimenting, with weekly smoking as the strongest predictor (OR 8.41; 95% CI 4.00–17.68). However, participating in team sports was not statistically significantly associated with e-cigarette experimentation more than twice at follow-up. The strongest predictors for follow-up smoking of more than 50 cigarettes (Table 3) were baseline daily, weekly and occasional smoking, drunkenness at least once, and daily energy drink use.

### 3.2. Predictors among Girls

For girls, the predictors of e-cigarette experimentation were rather the same as for boys, with daily (OR 19.88; 95% CI 8.05–49.13), weekly (OR 15.52; 95% CI 5.66–42.60) and occasional conventional smoking (OR 15.18; 95% CI 7.78–29.62) having the strongest associations (Table 4). Drunkenness at least once and energy drink use were also significant predictors for e-cigarette experimentation. Of leisure activities, only music, art or club activities were statistically significantly and negatively associated with e-cigarette experimentation. In multivariate analyses, conventional daily (OR 11.19; 95% CI 3.41–36.66), weekly (OR 6.57; 95% CI 1.99–21.70) and occasional smoking (OR 6.00; 95% CI 2.82–12.77) remained as the strongest predictors. Academic achievement and parents’ conventional smoking were statistically significantly associated with e-cigarette experimentation in both models. The predictors for smoking experimentation were fairly similar but mainly stronger compared to e-cigarette experimentation, with drunkenness at least once as the strongest predictor in both models (OR in Multivariate model: 7.15; 95% CI: 3.84–13.33).

The most significant predictors for girls’ e-cigarette experimentation more than twice were daily (OR 13.58; 95% CI 6.21–29.68) and occasional conventional smoking (OR 11.12; 95% CI 5.89–21.00), daily energy drink use (OR 9.98; 95% CI 5.16–19.30) and drunkenness at least once (OR 6.20; 95% CI 4.24–9.04) (Table 5). The predictors for girls’ smoking more than 50 cigarettes were quite similar to e-cigarette experimentation engaged in more than twice, except for leisure activities, which were all statistically significantly and negatively associated with smoking more than 50 cigarettes. Of academic achievement and socioeconomic and family background variables, academic achievement was the strongest predictor for both e-cigarette experimentation more than twice and for smoking more than 50 cigarettes. Parents’ conventional smoking was a statistically significant predictor for girls’ smoking more than 50 cigarettes, which was different from e-cigarette experimentation more than twice and from boys’ experimentation.

## 4. Discussion

We studied the predictors for adolescent e-cigarette experimentation and compared them with those for conventional cigarette smoking. The strongest predictors of e-cigarette experimentation were other addictive behaviors: cigarette smoking, drunkenness and the use of energy drinks. Excellent academic achievement protected from e-cigarette experimentation, while socioeconomic background was not important. Parents’ smoking also increased the risk for e-cigarette experimentation and for smoking, slightly more among girls than among boys. The predictors for experimenting with e-cigarettes and for experimenting with them more than twice were mostly similar. The exception was participation in team sports, which predicted e-cigarette experimentation among boys but not among girls and not for experimenting more than twice. The predictors for e-cigarettes and conventional cigarettes were mostly similar but the associations were slightly stronger for the latter.

Compared to the previous cross-sectional studies on e-cigarette correlates, we could confirm conventional cigarette smoking [4,5,10,11,12,13,14,15,16,17,18,19,20,21] and alcohol use [4,11,14,22] as predictors of e-cigarette experimentation in a longitudinal setting, although we used drunkenness and not lesser alcohol use as a predictor. Compared to the only one previous longitudinal study from Germany [37], we could confirm parental smoking as a risk factor for adolescent e-cigarette experimentation, but in contradiction to that study, we also found conventional cigarette smoking to be a risk factor. Previously, the predictors for e-cigarette experimentation have not been studied separately for boys and girls in longitudinal settings. When comparing predictors for e-cigarettes and conventional smoking, our findings coincided with those of Hanewinkel and Isensee [37]: the predictors were quite similar but not entirely the same. We discovered that the predictors were slightly stronger for conventional smoking compared to e-cigarette use. Wills et al. [41] also found out in their cross-sectional study that conventional cigarette smokers and dual users were higher on risk status, i.e., they were the most prone to problem behavior, while e-cigarette-only users had an intermediate risk status compared to non-users.

According to our results, participation in team sports at the age of 12 to 13 was a risk factor for boys’ e-cigarette experimentation at the age of 15 to 16. This result can be compared with a study of Mattila et al. [39], which showed that participation in team sports (e.g., ice hockey, football) was associated with snus use (Swedish moist snuff type) and with dual use of cigarettes and snus [39]. According to Social cognitive theory [42] and the Social norms approach [43], individual behavior is considerably determined by social norms. The social environment of team sports may be even more peer-oriented than other social environments, which can promote e-cigarette experimentation. This phenomenon has been discovered for snus use among ice-hockey players in Sweden [44]. Nicotine might improve concentration and performance of certain tasks [45], and physically active adolescents may find e-cigarettes less harmful to health and to oxygen intake compared to conventional cigarettes. However, the association between team sports and e-cigarette experimentation more than twice was not statistically significant, suggesting that while the social environment promotes e-cigarette experimentation, these adolescents may well not use e-cigarettes later on.

In this study, we were not able to reveal the reasons and motives behind the association between smoking at baseline and experimenting with e-cigarettes at follow-up. Did the adolescents try e-cigarettes to quit smoking? In our previous study, adolescents reported experimenting with e-cigarettes as they wanted to try something new, and because their friends started to use e-cigarettes [18]. Similar reasons have been reported also in other studies [4,11,46]. Only about one in ten adolescents reported using e-cigarettes to quit smoking in 2015 [18]. In a U.S. longitudinal study among adolescents [36], e-cigarette experimentation to quit smoking predicted continued e-cigarette use. Other such factors were e-cigarettes’ low cost and not smelling bad, and the ability to use them anywhere [36]. Nicotine addiction may explain this finding. There is still controversy over whether or not e-cigarettes are effective in smoking cessation, but according to a qualitative study, adolescents did not find e-cigarettes successful in quitting smoking [47].

Sensation-seeking behavior, as seeking out novel and exciting stimuli [48], has been found to be a predictor for e-cigarette experimentation [37]. Our results also show that different substance abuses are interrelated: strong predictors for e-cigarette experimentation were conventional cigarette smoking, drunkenness and energy drink use. It would be interesting in the future to study which product is the first experiment and which is the second experiment and so on, or if it is simply a matter of availability and opportunity.

In previous studies, adolescents have been asked if they have experimented with and used e-cigarettes with non-nicotine or nicotine-containing e-liquids [10,18]. However, adolescents do not always know whether the e-cigarette they used contained nicotine or not. In 2015, one in five Finnish adolescents, who had tried e-cigarettes, did not know about the contents of the e-liquid [18]. In our present study, we combined the groups experimenting with different e-liquids, as a large proportion of the adolescents had tried both. We conducted the analyses also separately according to the type of the e-liquid used and the results were very much the same and of the same magnitude (not shown in tables). Most of the adolescents had tried nicotine-containing e-cigarettes, which raises concern as it may increase the risk for nicotine dependency, and nicotine may have a long-lasting effect on adolescents’ developing brains [49].

The students had tried more frequently conventional cigarettes compared to e-cigarettes. Reported e-cigarette experimentation among boys in this study in 2014 was on a higher level (43.3%) compared to results of 16-year-olds boys in Finnish nationwide data from years 2013 and 2015 [18]: in 2013, 28.5% had tried e-cigarettes, and in 2015, 40.6%. Among 16-year-olds girls, however, the prevalence was 20.2% in 2013 and 31.5% in 2015 [18], which is in line with the prevalence from this study (25.6%). This survey was conducted in the metropolitan area of Helsinki, and the students do not represent all Finnish adolescents. It is possible that urban youth is more susceptible to new products, e.g., due to better availability and visibility of the products. The regional differences in adolescent e-cigarette use are worth studying more.

This study has also some limitations. The students in the sample in analyses had tried smoking less often and had slightly better academic achievement than the students who completed the baseline survey but not the follow-up survey. This means that the attrition contained more students who had tried smoking and more those with poorer academic achievement, which may have some effect on the associations. However, gender distribution did not differ statistically between the sample and the attrition, nor did the distribution of parental education. We used data reported by the adolescents themselves. Health-compromising behavior, like smoking and e-cigarette use, may have been underreported (or over reported) due to the desire to answer in a socially acceptable way in classrooms [50]. This may lead to under (or over) estimation of e-cigarette experimentation and smoking. However, adolescents’ self-reporting of conventional cigarette smoking has been reported to be good [51,52,53], which might be transferable to the self-report of e-cigarette use. The strength of our study is that the number of respondents is large. Due to a large study population, we were able to study the risk factors gender-stratified and still have nearly 3000 adolescents in each group. We were also able to study a great variety of predictors found in cross-sectional studies as correlates to e-cigarettes.

## 5. Conclusions 

To conclude, adolescent e-cigarette experimentation is strongly predicted by conventional cigarette smoking and by other addictive behaviors. Of socioeconomic and family background variables, adolescent’s low academic achievement is the strongest predictor of e-cigarette experimentation. The risk factors are mainly similar for e-cigarettes and conventional cigarettes, and for boys and girls. This means that prevention of this health-damaging behavior can follow similar models. In team sports, banning the use of e-cigarettes in all team activities for both the youth and coaches can be recommended. As many adolescents seek sensations, the availability of harmful substances (i.e., tobacco, alcohol, e-cigarettes) for minors should be made as difficult as possible.

## Figures and Tables

**Table 1:** Descriptive statistics of the study population by gender, %.

Predictor|Gender	Boys (*N* = 2871), % (*N*)	Girls (*N* = 2871), % (*N*)
Not tried e-cigarettes at follow-up	56.7 (1533)	74.4 (2079)
Tried e-cigarette once or twice at follow-up	21.5 (582)	17.4 (486)
Tried e-cigarettes 20 times or less at follow-up	6.7 (180)	4.5 (127)
Tried e-cigarettes more than 20 times at follow-up	15.2 (411)	3.6 (101)
Not tried smoking at follow-up	50.0 (1351)	57.7 (1609)
Tried smoking (1–50 cigarettes) at follow-up	32.0 (865)	29.4 (820)
Smoked more than 50 cigarettes at follow-up	18.0 (485)	12.9 (360)
**Addictive behavior at baseline**		
**Smoking**		
Never tried	78.2 (2166)	85.1 (2407)
Tried but does not smoke	17.1 (474)	10.9 (309)
Occasionally	2.3 (65)	1.9 (53)
Weekly	1.2 (34)	0.8 (24)
Daily	1.1 (31)	1.2 (34)
**Has been drunk at least once**	8.1 (223)	6.3 (179)
**Energy drink use**		
Never	45.3 (1258)	69.7 (1974)
Occasionally	48.1 (1336)	28.4 (805)
Daily	6.5 (181)	1.9 (54)
**Leisure activities at baseline**		
**Team sports**	44.2 (1268)	26.9 (771)
**Individual sports**	21.7 (622)	18.4 (529)
**Music, art or club activities**	20.0 (575)	31.8 (914)
**Academic achievement at baseline**		
Excellent	21.1 (595)	28.1 (797)
Good	43.0 (1210)	45.3 (1286)
Satisfactory	29.1 (818)	21.7 (615)
Poor	6.8 (192)	5.0 (141)
**Socioeconomic and family background at baseline**		
**Family structure not intact**	29.2 (834)	28.4 (810)
**Parents’ education**		
University degree (>15 years of education)	34.8 (849)	34.0 (849)
Matriculations examination/A-levels (12 years)	28.7 (700)	29.4 (735)
Vocational education and training (12 years)	21.1 (515)	20.6 (514)
Comprehensive school (9 years)	15.4 (376)	16.0 (401)
**Parents’ smoking**		
Neither of them smokes	50.0 (1378)	49.6 (1402)
Mother/father has smoked	21.7 (597)	21.4 (604)
Mother/father smokes	21.2 (584)	21.3 (601)
Both of them smoke	7.2 (198)	7.7 (217)

**Table 2:** Odds ratios (OR) and the 95% confidence interval of multilevel binary logistic regression for follow-up e-cigarette and smoking experimentation by baseline predictors among boys.

	Tried E-Cigarette	Tried E-Cigarette	Tried Smoking	Tried Smoking
	Bivariate	Multivariate	Bivariate	Multivariate
	Model *	Model ^¥^	Model *	Model ^¥^
Baseline Predictor	OR (95% CI)	OR (95% CI)	OR (95% CI)	OR (95% CI)
**Addictive behavior**				
**Smoking** (ref = Never tried)				
Tried but does not smoke	**4.74** (3.78–5.94)	**3.33** (2.54–4.36)	n.a.	n.a.
Occasionally	**15.58** (7.05–34.45)	**7.02** (2.85–17.31)	n.a.	n.a.
Weekly	**9.60** (3.64–25.30)	**5.04** (1.64–15.50)	n.a.	n.a.
Daily	**61.12** (8.30–450.0)	**19.26** (2.51–147.7)	n.a.	n.a.
**Has been drunk** (ref = Never)				
At least once	**7.08** (4.92–10.21)	**2.33** (1.49–3.65)	**15.49** (9.09–26.40)	**9.41** (5.23–16.95)
**Energy drink use** (ref = Never)				
Occasionally	**3.27** (2.76–3.88)	**2.23** (1.82–2.73)	**3.82** (3.23–4.52)	**2.95** (2.43–3.58)
Daily	**6.70** (4.63–9.69)	**3.16** (1.92–5.19)	**10.67** (6.91–16.46)	**6.27** (3.65–10.77)
**Leisure activities**				
**Team sports** (ref = No)				
Yes	**1.43** (1.23–1.67)	**1.90** (1.55–2.32)	0.93 (0.80–1.09)	n.s.
**Individual sports** (ref = No)				
Yes	**0.80** (0.66–0.96)	1.20 (0.95–1.52)	0.86 (0.72–1.03)	n.s.
**Music, art or club activities** (ref = No)				
Yes	**0.63** (0.52–0.77)	0.82 (0.64–1.04)	**0.74** (0.62–0.90)	1.02 (0.80–1.28)
**Academic achievement** (ref = Excellent)				
Good	**1.67** (1.35–2.07)	**1.32** (1.02–1.71)	**1.94** (1.57–2.40)	**1.76** (1.37–2.27)
Satisfactory	**2.39** (1.89–3.00)	**1.38** (1.03–1.86)	**3.25** (2.58–4.10)	**2.14** (1.60–2.87)
Poor	**3.63** (2.54–5.19)	**2.13** (1.31–3.46)	**4.27** (2.96–6.17)	**2.39** (1.48–3.86)
**Socioeconomic and family background**				
**Family structure** (ref = Intact family)				
Other family type	**1.37** (1.16–1.62)	0.95 (0.76–1.18)	**1.80** (1.52–2.13)	1.18 (0.95–1.47)
**Parents’ education** (ref = University)				
Matriculations	1.19 (0.96–1.46)	1.12 (0.87–1.42)	0.99 (0.81–1.22)	0.81 (0.64–1.03)
examination/A-levels				
Vocational education	**1.45** (1.15–1.82)	1.14 (0.86–1.50)	1.26 (1.00–1.58)	0.76 (0.58–1.00)
and training				
Comprehensive school	**1.40** (1.09–1.81)	1.11 (0.81–1.50)	**1.49** (1.15–1.92)	0.94 (0.69–1.27)
**Parents’ smoking** (ref = Neither of them smokes)				
Mother/father has smoked	**1.62** (1.33–1.98)	**1.38** (1.08–1.77)	**1.71** (1.40–2.10)	**1.48** (1.16–1.89)
Mother/father smokes	**1.78** (1.46–2.18)	**1.60** (1.23–2.07)	**2.06** (1.68–2.52)	**1.72** (1.33–2.22)
Both of them smoke	**1.93** (1.41–2.63)	1.08 (0.71–1.63)	**2.99** (2.15–4.16)	**1.83** (1.21–2.77)

* Bivariate model: Bivariate logistic regression, 2-level analyses, school as the random effect; ^¥^ Multivariate model: Multivariate logistic regression, includes all statistically significant variables from Bivariate model, 2-level analyses, school as the random effect; Note. Odds ratio (OR) is given in boldface when it indicates a statistically significant (*p* < 0.05) difference from the odds of the reference category; n.s. = not significant in Bivariate model; n.a. = not applicable.

**Table 3:** Odds ratios (OR) and the 95% confidence interval of multilevel binary logistic regression for follow-up e-cigarette experimentation more than twice and smoking more than 50 cigarettes by baseline predictors among boys.

	Tried E-Cigarette	Tried E-Cigarette	Smoked > 50	Smoked > 50
	>Twice	>Twice	Cigarettes	Cigarettes
	Bivariate	Multivariate	Bivariate	Multivariate
	Model *	Model ^¥^	Model *	Model ^¥^
Baseline Predictor	OR (95% CI)	OR (95% CI)	OR (95% CI)	OR (95% CI)
**Addictive behavior**				
**Smoking** (ref = Never tried)				
Tried but does not smoke	**3.72** (2.97–4.66)	**2.36** (1.83–3.04)	**6.04** (4.75–7.70)	**4.11** (3.07–5.50)
Occasionally	**7.92** (4.72–13.29)	**3.23** (1.77–5.93)	**14.69** (8.59–25.10)	**6.41** (3.34–12.31)
Weekly	**8.41** (4.00–17.68)	**3.89** (1.72–8.83)	**37.10** (14.91–92.34)	**18.45** (6.51–52.29)
Daily	**8.08** (3.94–16.57)	**2.38** (1.05–5.38)	**49.54** (18.75–130.9)	**22.91** (7.39–71.08)
**Has been drunk** (ref = Never)				
At least once	**4.96** (3.71–6.63)	**2.16** (1.51–3.10)	**7.45** (5.52–10.05)	**2.11** (1.41–3.14)
**Energy drink use** (ref = Never)				
Occasionally	**3.69** (2.96–4.61)	**2.61** (2.05–3.33)	**3.32** (2.61–4.24)	**1.68** (1.25–2.25)
Daily	**5.60** (3.86–8.12)	**2.36** (1.50–3.70)	**7.10** (4.84–10.40)	**1.80** (1.06–3.05)
**Leisure activities**				
**Team sports** (ref = No)				
Yes	1.12 (0.93–1.35)	n.s.	**0.78** (0.64–0.96)	0.95 (0.73–1.24)
**Individual sports** (ref = No)				
Yes	**0.73** (0.58–0.92)	0.88 (0.68–1.15)	**0.73** (0.56–0.94)	1.06 (0.77–1.46)
**Music, art or club activities** (ref = No)				
Yes	**0.61** (0.47–0.78)	**0.74** (0.56–0.99)	0.80 (0.62–1.04)	n.s.
**Academic achievement** (ref = Excellent)				
Good	**1.45** (1.10–1.92)	1.12 (0.82–1.53)	**2.09** (1.48–2.94)	**2.07** (1.35–3.16)
Satisfactory	**2.40** (1.80–3.20)	1.37 (0.99–1.90)	**3.89** (2.76–5.50)	**2.58** (1.65–4.04)
Poor	**4.37** (2.96–6.43)	**2.09** (1.33–3.26)	**5.52** (3.54–8.60)	**2.75** (1.51–4.99)
**Socioeconomic and family background**				
**Family structure** (ref = Intact family)				
Other family type	**1.55** (1.27–1.88)	1.23 (0.98–1.56)	**2.00** (1.63–2.46)	1.22 (0.92–1.62)
**Parents’ education** (ref = University)				
Matriculations	1.16 (0.90–1.50)	n.s.	0.97 (0.73–1.29)	0.79 (0.56–1.11)
examination/A-levels				
Vocational education	1.28 (0.98–1.69)	n.s.	1.21 (0.90–1.64)	0.81 (0.56–1.16)
and training				
Comprehensive school	**1.36** (1.00–1.84)	n.s.	**1.57** (1.15–2.15)	1.07 (0.73–1.58)
**Parents’ smoking** (ref = Neither of them smokes)				
Mother/father has smoked	**1.71** (1.35–2.16)	1.22 (0.94–1.60)	**1.89** (1.45–2.46)	1.17 (0.84–1.64)
Mother/father smokes	**1.54** (1.21–1.96)	1.06 (0.80–1.40)	**2.10** (1.62–2.72)	**1.42** (1.01–2.00)
Both of them smoke	**1.79** (1.25–2.54)	0.87 (0.57–1.33)	**3.26** (2.29–4.64)	1.43 (0.86–2.35)

* Bivariate model: Bivariate logistic regression, 2-level analyses, school as the random effect; ^¥^ Multivariate model: Multivariate logistic regression, includes all statistically significant variables from Bivariate model, 2-level analyses, school as the random effect; Note. Odds ratio (OR) is given in boldface when it indicates a statistically significant (*p* < 0.05) difference from the odds of the reference category; n.s. = not significant in Bivariate model; n.a. = not applicable.

**Table 4:** Odds ratios (OR) and the 95% confidence interval of multilevel binary logistic regression for follow-up e-cigarette and smoking experimentation by baseline predictors among girls.

	Tried E-Cigarette	Tried E-Cigarette	Tried Smoking	Tried Smoking
	Bivariate	Multivariate	Bivariate	Multivariate
	Model *	Model ^¥^	Model *	Model ^¥^
Baseline Predictor	OR (95% CI)	OR (95% CI)	OR (95% CI)	OR (95% CI)
**Addictive behavior**				
**Smoking** (ref = Never tried)				
Tried but does not smoke	**6.82** (5.26–8.85)	**3.66** (2.66–5.02)	n.a.	n.a.
Occasionally	**15.18** (7.78–29.62)	**6.00** (2.82–12.77)	n.a.	n.a.
Weekly	**15.52** (5.66–42.60)	**6.57** (1.99–21.70)	n.a.	n.a.
Daily	**19.88** (8.05–49.13)	**11.19** (3.41–36.66)	n.a.	n.a.
**Has been drunk** (ref = Never)				
At least once	**6.95** (4.96–9.73)	1.49 (0.93–2.39)	**17.51** (10.06–30.48)	**7.15** (3.84–13.33)
**Energy drink use** (ref = Never)				
Occasionally	**4.31** (3.56–5.21)	**2.42** (1.91–3.06)	**6.16** (5.11–7.43)	**4.34** (3.50–5.40)
Daily	**6.45** (3.62–11.51)	1.70 (0.78–3.71)	**9.62** (4.76–19.45)	**4.57** (1.86–11.21)
**Leisure activities**				
**Team sports** (ref = No)				
Yes	0.98 (0.81–1.19)	n.s.	1.01 (0.85–1.20)	n.s.
**Individual sports** (ref = No)				
Yes	0.83 (0.66–1.04)	n.s.	**0.80** (0.65–0.97)	1.05 (0.82–1.34)
**Music, art or club activities** (ref = No)				
Yes	**0.72** (0.59–0.87)	0.89 (0.70–1.12)	**0.66** (0.56–0.79)	0.92 (0.75–1.13)
**Academic achievement** (ref = Excellent)				
Good	**2.22** (1.75–2.82)	**1.71** (1.29–2.25)	**2.17** (1.78–2.64)	**1.74** (1.38–2.20)
Satisfactory	**3.06** (2.34–4.01)	**1.53** (1.09–2.16)	**3.93** (3.12–4.96)	**2.54** (1.89–3.40)
Poor	**3.99** (2.64–6.03)	1.69 (0.96–2.98)	**5.64** (3.78–8.42)	**3.89** (2.22–6.82)
**Socioeconomic and family background**				
**Family structure** (ref = Intact family)				
Other family type	**1.47** (1.22–1.78)	1.00 (0.79–1.27)	**1.80** (1.52–2.13)	1.21 (0.98–1.50)
**Parents’ education** (ref = University)				
Matriculations	1.18 (0.93–1.50)	1.16 (0.88–1.53)	1.16 (0.94–1.43)	1.03 (0.81–1.30)
examination/A-levels				
Vocational education	**1.52** (1.18–1.98)	1.00 (0.73–1.37)	**1.57** (1.24–1.97)	0.92 (0.69–1.21)
and training				
Comprehensive school	1.31 (0.99–1.74)	0.80 (0.57–1.13)	**1.60** (1.25–2.05)	0.75 (0.55–1.02)
**Parents’ smoking** (ref = Neither of them smokes)				
Mother/father has smoked	**1.86** (1.48–2.34)	**1.32** (1.00–1.74)	**1.94** (1.59–2.37)	**1.42** (1.11–1.80)
Mother/father smokes	**2.24** (1.79–2.81)	**1.54** (1.16–2.05)	**2.52** (2.06–3.08)	**1.75** (1.36–2.27)
Both of them smoke	**3.30** (2.41–4.51)	**1.80** (1.20–2.69)	**4.06** (2.99–5.52)	**2.05** (1.39–3.02)

* Bivariate model: Bivariate logistic regression, 2-level analyses, school as the random effect; ^¥^ Multivariate model: Multivariate logistic regression, includes all statistically significant variables from Bivariate model, 2-level analyses, school as the random effect; Note. Odds ratio (OR) is given in boldface when it indicates a statistically significant (*p* < 0.05) difference from the odds of the reference category; n.s. = not significant in Bivariate model; n.a. = not applicable.

**Table 5:** Odds ratios (OR) and the 95% confidence interval of multilevel binary logistic regression for follow-up e-cigarette experimentation more than twice and smoking more than 50 cigarettes by baseline predictors among girls.

	Tried E-Cigarette	Tried E-Cigarette	Smoked > 50	Smoked > 50
	>Twice	>Twice	Cigarettes	Cigarettes
	Bivariate	Multivariate	Bivariate	Multivariate
	Model *	Model ^¥^	Model *	Model ^¥^
Baseline Predictor	OR (95% CI)	OR (95% CI)	OR (95% CI)	OR (95% CI)
**Addictive behavior**				
**Tried smoking** (ref = Never tried)				
Tried but does not smoke	**4.87** (3.46–6.85)	**2.57** (1.66–3.97)	**8.44** (6.32–11.26)	**2.99** (2.08–4.30)
Occasionally	**11.12** (5.89–21.00)	**3.17** (1.36–7.39)	**12.37** (6.81–22.45)	**2.67** (1.25–5.73)
Weekly	2.44 (0.69–8.63)	0.90 (0.22–3.70)	**88.77** (25.66–307.2)	**21.71** (5.56–84.74)
Daily	**13.58** (6.21–29.68)	**5.08** (1.84–14.00)	**62.16** (24.78–155.9)	**19.77** (5.85–66.79)
**Has been drunk** (ref = Never)				
At least once	**6.20** (4.24–9.04)	**2.10** (1.20–3.67)	**11.69** (8.34–16.39)	**2.01** (1.23–3.30)
**Energy drink use** (ref = Never)				
Occasionally	**3.13** (2.33–4.21)	**1.92** (1.32–2.79)	**6.78** (5.27–8.73)	**3.42** (2.49–4.70)
Daily	**9.98** (5.16–19.30)	**3.94** (1.66–9.32)	**13.46** (7.35–24.65)	1.87 (0.75–4.63)
**Leisure activities**				
**Team sports** (ref = No)				
Yes	1.05 (0.77–1.43)	n.s.	**0.75** (0.57–0.98)	0.77 (0.54–1.09)
**Individual sports** (ref = No)				
Yes	1.01 (0.70–1.44)	n.s.	**0.60** (0.43–0.84)	0.85 (0.56–1.31)
**Music, art or club activities** (ref = No)				
Yes	0.79 (0.58–1.07)	n.s.	**0.63** (0.48–0.81)	0.83 (0.59–1.17)
**Academic achievement** (ref = Excellent)				
Good	**3.00** (1.92–4.70)	**2.39** (1.43–3.98)	**4.24** (2.75–6.55)	**2.98** (1.77–5.01)
Satisfactory	**3.67** (2.26–5.96)	**2.15** (1.20–3.85)	**8.31** (5.31–12.99)	**3.94** (2.25–6.88)
Poor	**5.21** (2.73–9.95)	**2.53** (1.12–5.70)	**13.02** (7.50–22.62)	**4.23** (2.01–8.89)
**Socioeconomic and family background**				
**Family structure** (ref = Intact family)				
Other family type	**1.44** (1.07–1.93)	0.92 (0.64–1.32)	**1.94** (1.54–2.45)	1.08 (0.79–1.47)
**Parents’ education** (ref = University)				
Matriculations	1.40 (0.94–2.08)	1.40 (0.90–2.18)	1.27 (0.91–1.78)	1.15 (0.76–1.73)
examination/A-levels				
Vocational education and training	**2.05** (1.36–3.08)	1.49 (0.93–2.38)	**1.99** (1.41–2.80)	1.05 (0.68–1.62)
Comprehensive school	1.21 (0.74–1.97)	0.89 (0.52–1.55)	**2.00** (1.39–2.87)	0.98 (0.62–1.55)
**Parents’ smoking** (ref = Neither of them smokes)				
Mother/father has smoked	1.44 (0.98–2.10)	1.04 (0.67–1.61)	**2.77** (2.01–3.81)	**1.81** (1.22–2.69)
Mother/father smokes	**2.04** (1.43–2.90)	1.17 (0.75–1.83)	**3.76** (2.78–5.10)	**1.87** (1.25–2.79)
Both of them smoke	**2.82** (1.79–4.44)	1.27 (0.72–2.24)	**6.31** (4.34–9.17)	**2.40** (1.46–3.94)

* Bivariate model: Bivariate logistic regression, 2-level analyses, school as the random effect; ^¥^ Multivariate model: Multivariate logistic regression, includes all statistically significant variables from Bivariate model, 2-level analyses, school as the random effect; Note. Odds ratio (OR) is given in boldface when it indicates a statistically significant (*p* < 0.05) difference from the odds of the reference category; n.s. = not significant in Bivariate model; n.a. = not applicable.

## Appendix A

**Table A1:** Names of variables, questions, answering options and categories of predictors.

Variable Name	Question in Questionnaire	Answering Options	Predictor Categories
**Smoking**	(1) Have you ever smoked?	I have never tried	Never tried
	If you have, how many		Tried but do not smoke
	cigarettes have you smoked		
	altogether until now?		
	(2) Which of the following		
	options best describes	Less than once a week	Occasionally
	your current smoking?		Weekly
	
		Once a day or more often	Daily
**Has been**	Have you sometimes drunk	Never	No
**drunk**	so much alcohol that		Yes
	you have gotten drunk?		
	
**Energy drink**	How often do you		Daily
**use**	drink energy drinks ?		
			Occasionally
	
		Not at all	Never
	What are you into at least		
	once a week…?		
**Team sports**	Team sports (e.g., football, floorball)	Tick, if yes	No/Yes
**Individual**	Individual sports (e.g.,	Tick, if yes	No/Yes
**sports**	athletics, skiing, tennis, judo)		
**Music, art or**	Music (e.g., lessons, practicing		No/Yes
**club activities**	at home, music theory)		
	Art (e.g., painting,		
	making videos)		
	Club activities (e.g., scouting,		
	chess, 4-H or similar clubs)		
**Academic**	Your grades in the following	Open space for answer	Calculated mean:
**achievement**	theoretical subjects?	Scale for grades: 4–10	9.0–10.0 = Excellent
	Mother tongue		8.0–8.9 = Good
	Mathematics		7.0–7.9 = Satisfactory
	First foreign (A1-)language		4.0–6.9 = Poor
	History		
	Chemistry		
**Family**	Who do you live with?	With both of my parents	Intact family
**structure**			Other family type
	
	
	
	
	
	
	
**Parents’**	What kind of education	Comprehensive school	Comprehensive school
**education**			(9 years of education)
(Combined for	do your parents have?	Vocational degree	Vocational education
			and training (12 years)
the highest	(Separately for	Matriculation exam	Matriculations examina-
			tion/A-levels (12 years)
education	mother and father)	University degree	University degree
			(>15 years)
of either parent)		I have no mother/father	(Missing)
**Parents’**	Have your parents smoked	Has never smoked	Neither of them smokes
**smoking**	during your lifetime?	Has smoked but quitted	Mother/father has smoked
	(Separately for	Smokes nowadays	Mother/father smokes
	mother and father)		Both of them smoke
		I have no mother/father	(Missing)
